# The Scale of Self-Efficacy Expectations of Adherence to Antiretroviral Treatment: A Tool for Identifying Risk for Non-Adherence to Treatment for HIV

**DOI:** 10.1371/journal.pone.0147443

**Published:** 2016-02-19

**Authors:** Maria de Lourdes Drachler, Carlos Wietzke Drachler, Luciana Barcellos Teixeira, José Carlos de Carvalho Leite

**Affiliations:** 1 Secretaria da Saúde do Estado do Rio Grande do Sul, Governo do Estado do Rio Grande do Sul, Av. Bento Gonçalves 3722, CEP: 90650–001, Porto Alegre, RS, Brazil; 2 Rede Governo Colaborativo em Saúde, Universidade Federal do Rio Grande do Sul, Av. João Pessoa 155, CEP: 90040–001, Porto Alegre, RS, Brazil; 3 Programa de Pós-Graduação em Saúde Coletiva, Escola de Enfermagem, Universidade Federal do Rio Grande do Sul, Rua São Manoel 963, CEP: 90.620–110, Porto Alegre, RS, Brazil; 4 Mestrado Profissional em Saúde e Desenvolvimento Humano, Centro Universitário La Salle, Av. Victor Barreto, 2288, CEP: 92010–000, Canoas, RS, Brazil; International AIDS Vaccine Initiative, UNITED STATES

## Abstract

**Background:**

Identification of risk for non-adherence to treatment is a challenge for personalized care for people living with HIV. Standardized questionnaires of patients’ expectations of their capability to overcome obstacles for treatment adherence may be used as a pre-screening for risk identification. A scale of self-efficacy expectations of adherence to antiretroviral treatment (SEA-ART scale) was previously developed. This study assesses the scale validity in predicting non-adherence to ART in adults living with HIV.

**Methods and Findings:**

A prospective cohort study applied a 21-item SEA-ART scale to 275 adults in ART treatment at an outpatient public service for HIV in Southern Brazil. ART medications taken were assessed at one-month follow-up; ART adherence was devised as an intake of 95% and more of the prescribed medication. A SEA-ART score was calculated by adding up the scores of all items. Multivariable logistic regression and the Area Under the Receiver-Operating-Characteristic Curve (AUROC) were applied to examine the ability of the SEA-ART score to predict non-adherence at follow-up. The SEA-ART score varied from 21 to 105; mean 93.9; median 103.0. Non-adherence was 30.3% (n = 81/267). The odds of non-adherence was 8% lower for each unit increase of the SEA-ART score; after adjustment for age, sex, formal education and time in treatment (OR = 0.92; 95%CI 0.90–0.95; LRT for linear trend, p = 0.002). The AUROC was 0.80 (95%CI 0.73–0.87; p<0.001). The SEA-ART optimal cut-off value was 101, providing a sensitivity of 76.5%, a specificity of 73.1%, a positive predictive value of 55.4% and a negative predictive value of 87.7%. There was no evidence of difference in sensitivity, and specificity among groups organized by age, gender, formal education and time in treatment.

**Conclusions:**

The SEA-ART scale appears to have a good capacity to discriminate between adherents and non-adherents at one-month follow-up. Further studies should confirm these results in other populations.

## Introduction

There is a strong interest in identification of risk for non-adherence to antiretroviral treatment (ART) for HIV to promote therapy effectiveness. Positive health outcomes usually require systematic use of 90 to 95% of the recommended treatment [1,2,3], but approximately 30% (5.0 to 67.0%) of the patients do not comply with the therapy, even in countries with universal access to ART, as in Brazil [4,5,6].

Scales developed from the application of standardized questionnaires of self-efficacy expectations for adherence to ART may support risk identification. Patients´ expectations of their self-efficacy for adherence to treatment have been considered a major motivational factor for treatment adherence. Applied to health behaviours, the expectations of self-efficacy is the person´s anticipation of his/her own capability of adopting a particular health-promoting behaviour by personal action throughout a given period of time in the future [7], such as following the antiretroviral treatment recommendations by personal commitment during the next month. Such belief on own efficacy to overcome obstacles and achieve goals set have been shown to predict the initiation and sustenance of behaviour change and adherence to treatment of various conditions for example, maintenance haemodialysis, tobacco smoking. A low and ineffective sense of personal control have been shown to be associated with non-adherence to treatment [8,9,10].

The Scale of Self-efficacy Expectations of Adherence to Antiretroviral Treatment (SEA-ART) assesses patients’ expectations of their own ability to follow the antiretroviral prescription in 21 high-risk situations for non-adherence to ART. The SEA-ART scale was developed by content analysis of interviews with adults non-adherents and adherents to antiretroviral treatment in Southern Brazil [11]. The scale is organized in areas: (a) *environmental circumstances and treatment scheme* which require great attention and organization to follow the medical prescription, (b) *unsupportive social relationships* which may decrease confidence in treatment and (c) e*motional and physiological states*, including negative experiences with the treatment, negative affect and low concern with the illness. The SEA-ART scale have shown to be associated with treatment adherence in cross-sectional studies in different populations: adults in advanced clinical stages of AIDS attending a specialised day-hospital facility [12], children and adolescents [13] and adults [14,15] in outpatient public services for people living with HIV in Brazil.

The cut-off point of the SEA-ART scale most likely to predict non-adherence to ART still needs to be established to increase the scale potential for personalized care. The probability of a scale correctly assessing the adherence status of a patient (negative and positive predictive value) depends on the prevalence of non-adherence in the population and on the scale ability to identify non-adherence (sensitivity) or adherence (specificity). This study identifies the cut-off point of the SEA-ART scale which is associated with the highest specificity and sensitivity in predicting non-adherence to ART in adults living with HIV in Southern Brazil.

## Material and Methods

### Participants and procedures

A one-month observational study of adherence to antiretroviral treatment for HIV was conducted in the specialised outpatient public health service, which was the reference for people living with HIV in the city of Pelotas and five other municipalities, with a catchment area of about 300,000 inhabitants in the Southern Brazilian state of Rio Grande do Sul.

The participant individuals were all adults (≥18 years) registered at the participant service who started antiretroviral treatment in the three years preceding the study, both service attenders and dropouts. The selected individuals were invited to take part of the study by a member of the health staff when attending at the service or during home visits. The data collection included two interviews with the patients conducted by a trained fieldworker usually at the health service, and clinical data collected from the patients´ records by a medical researcher.

The first interview assessed sociodemographic characteristics (age, sex and formal education in years of schooling) collected by closed questions and self-efficacy expectations of adherence to antiretroviral therapy assessed by the 21-item SEA-ART scale [11], presented in Table 1. For each item of the SEA-ART scale, the patients indicated on a Likert scale how confident they were that they would be able take the `HIV medication`during the next month, exactly as prescribed by the physician. The scale was coded: 1 = not at all confident; 2 = slightly confident; 3 = don´t know; 4 = moderately confident; and 5 = extremely confident. The second interview investigated names and doses of medications taken at one-month follow-up, by eliciting information about medications taken in the past 48 hours. The interviewer was not aware of the antiretroviral regimen prescribed. The current ART scheme of each patient and treatment effectiveness measured by undetected viral load (<80copies/ml) within six months after the self-reported adhesion were copied from the patient´s clinical file by a medical researcher.

**Table 1:** Participants’ socio-demographic characteristics and time in treatment.

Participant variables	Number of Participants	
	N	(%)
**Gender**		
Male	177	64.4
Female	98	35.6
**Age at first interview (years)**		
≤ 24	41	14,9
25 to 34	93	33,8
35 to 44	93	33,8
≥45	48	17,5
**Education (years of schooling)**		
≤ 4	106	38.5
5 to 7	80	29.1
≥ 8	89	32.4
**Time in treatment (months)**		
≤ 6.0	78	28.4
6.1–18.0	101	36.7
>18.0	96	34.9
**Total**	275	100

### Statistical analysis

#### Devising the SEA-ART total score at baseline

The SEA-ART items were scrutinized for inadequacy, defined by poor acceptability (missing data greater than 5%), endorsement frequencies greater than 80% in any of the response categories, and poor contribution to the scale (corrected-item-total correlations ≤ 0.30). Internal consistency reliability of the 21-item scale was measured by the Crombach-α index (ideally > 0.70). The score of self-efficacy expectations of adhesion to ART (SEA-ART total score) was calculated for each patient by adding up the scores of all items.

#### Devising self-reported adherence to ART at one-month follow-up

The percentage of adherence was calculated for each drug prescribed, dividing the number of pills reported to be taken by the number of pills prescribed. Adherence to ART scheme was calculated by the sum of the adherences to all drugs, divided by the number of drugs prescribed. Patients were organized in two groups: non-adherents and adherents to ART, with non-adherence defined as an intake of less than 95% of the prescribed medications.

#### Establishing the ability of the scale in predicting non-adherence to ART

Evidence of difference in means of self-efficacy expectations between non-adherents and adherents to ART was assessed by independent sample T-Test. Logistic regression was applied to model a linear trend of the odds ratio of non-adherence to ART for each unit increase in SEA-ART score, adjusted for age, gender and formal education.

A receiver operating characteristic (ROC) curve analysis was estimated to assess the effectiveness of the SEA-ART score to detect non-adherence to the antiretroviral treatment. The ROC curve was estimated for the whole sample and separately for groups defined by age, gender, formal education and time in treatment. The area under the ROC curve (AUROC) gives the probability of discriminating who is the non-adherent individual between two individuals randomly selected, one from each of two groups: non-adherents and adherents [16]. An AUROC ≥ 0.70 indicates good performance of the scale. Sensitivity and specificity of the SEA-ART overall total score were calculated. The Youden´s index-*J* [17] was applied to examine the accuracy of the SEA-ART scale, defined by the cut-off point of the scale which provides the highest specificity and sensitivity in predicting non-adherence. Sensitivity, specificity, positive and the negative predictive values were calculated for the whole sample and for groups organized by age, gender, formal education and time in antiretroviral treatment. Sensitivity was calculated as the percentage of individuals with SEA-ART score below or equal to the cut-off point, among those who were non-adherent to treatment; specificity was the percentage of SEA-ART score above the cut-off among adherent individuals; the positive predictive value was the percentage of non-adherence among individuals with SEA-ART below or equal to the cut-off, and the negative predictive value was the percentage of adherence among individuals with SEA-ART above the cut-off point of the scale.

The statistical analyses were conducted using the statistical package SPSS 22.0 [18]. A 5% statistical significance (p-value < 0.05) was previously established.

### Ethical information

This study was conformed according to the principles of the Declaration of Helsinki and ethical clearance were obtained from the Ethics Committee of the Federal University of Pelotas, Rio Grande do Sul, Brazil. All patients gave their written informed consent to participate and were free to refuse to participate in the study or to withdraw consent to participate at any time without compromising any aspect of their treatment.

## Results

There were 312 individuals who met the inclusion criteria; 88.1% (n = 275/312) took part at the first interview and 85.6% (n = 267/312), at the second interview. Two individuals refused to take part in the study; 35 were not found for the first interview; five died and one moved to another city during the follow-up period; two refused to give the follow-up interview. Participants’ socio-demographic characteristics and time in ART treatment are shown in Table 1. Most participants were men (64.4%), from 25 to 44 years old (66.8%), with low levels of schooling (≤ 4 years = 38.5%, 5 to 7 years = 29.1%) and time in ART treatment greater than six months (71.6%).

Non-adherence to ART was 30.3% (n = 81/267 patients) at one-month follow-up; 48% of the patients (n = 118/244) presented undetectable viral load (<80 copies/ml) within six months after the measurement of self-reported adhesion. Undetectable viral load was positively associated with self-reported adherence (χ^2^ for trend p-value = <0.001). The percentage of patients with undetectable viral load was 27% for adherence less than 80%, and it was 57% for both groups of adherence: 80–94% and ≥95% of the prescribed ART scheme.

### Devising the SEA-ART total score at baseline

Item analysis showed that non-response rate was less than 1% for all items and none of the items had endorsement frequencies greater than 80%, suggesting good acceptability of the items and reasonable distribution of item responses. The corrected-item-total correlations above 0.49 for the 21 items suggest that all items provide a reasonable contribution to the scale. The Crombach-α index of 0.95 indicates a high internal consistency reliability of the scale.

Low self-efficacy expectations varied among the 21 risk situations measured by the items of the SEA-ART scale (Table 2). Low efficacy expectations for adherence to ART were most common for experiences of ‘adverse effects of the treatment’ (item 21 = 25.8% of the patients), ‘being with people the patient don’t want to disclose the HIV condition’ (item 9 = 12.4%), ‘going from one place to another’ and ‘being with strangers’ at the time they should take the medication (item 7 = 8.0% and item 14 = 7.6%).

**Table 2:** Participants with low self-efficacy expectations of adherence to treatment in the risk situations for non-adherence measured by the SEA-ART scale^a^.

Risk situations for non-adherence to treatment	Participants with low efficacy expectations^b^	
How confident are you that you´d be able take the HIV medication during the next month, as prescribed by your doctor	n	(%)
**Emotional and physiological states**		
If I’m feeling well (item 1)	10	(3.6)
If my viral load is too small to show up in the blood test (item2)	9	(3.3)
If I’m depressed and felling down (item 3)	11	(4.0)
If I’m feeling ill (item 8)	7	(2.5)
If I’m nervous or irritable (item 11)	15	(5.5)
If the medicine is causing unwanted effects (item 21)	71	(25.8)
**Unsupportive social relationships**		
If I’m discriminated or rejected (item 4)	8	(2.9)
If I’m with people I don’t want to disclose I’m HIV positive (item 9)	34	(12.4)
If I don’t have my own doctor (item 12)	15	(5.5)
If I’m with an outsider (item 14)	21	(7.6)
If I’m with people who disbelieve in HIV treatment (item 20)	8	(2.9)
**Environmental circumstances and treatment scheme**		
If I’m busy or enjoying myself (item 5)	19	(6.9)
If I’m on holiday or at work (item 6)	11	(4.0)
If I’m going from one place to another. (item 7)	22	(8.0)
If I have to take lots of pills. (item 10)	15	(5.5)
If I must take medications many times a day. (item 1)	13	(4.7)
If I have a hard time swallowing pills. (item15)	16	(5.8)
If I’m in holidays or at weekends. (item 16)	5	(1.8)
If I have to change my eating or sleeping habits. ((item 17)	6	(2.2)
If the medicine tastes or smells badly. (item 18)	16	(5.8)
If I’m doing things out of my daily routines. (item 19)	14	(5.1)

^a^ SEA-ART scale—The scale of self-efficacy expectations of adherence to antiretroviral treatment

^b^ Low expectations include not at all and slightly confident in taking the medications as prescribed

### SEA-ART scale prediction of non-adherence to ART

The SEA-ART score at baseline varied from 21 to 105; mean 93.9. Non-adherence to ART was 30.3% (n = 81/267 patients) at one-month follow-up. Table 3 shows that the average SEA-ART score at the first interview was lower among the non-adherents compared to those who were adherent to treatment at one month follow-up (SEA-ART mean 76.0 and 101.7 units respectively; Independent sample *t*-test p<0.001). In logistic regression, the odds of non-adherence was 8% lower for each unit of increase in SEA-ART score, after adjustment for age in years, sex, formal education in years and time in treatment in months (Odds ratios = 0.92; 95% CI 0.90–0.95; LRT test for linear trend with increasing SEA-ART score, p = 0.002).

**Table 3:** Means of SEA-ART scale score at baseline by treatment status at one month follow-up and linear trends of Odds Ratios of non-adherence to treatment at follow-up for unit increase in SEA-ART score at baseline.

SEA-ART scale score^a^ at baseline			Odds Ratios^b^ of non-adherence at follow-up for unit increase in SEA-ART score at baseline		
	Mean	p-value^c^	OR	(95% CI)	p-value^d^
Non-adherents	76.0				
Adherents	101.7				
Total	93.9	<0.001			
			0.92	(0.90 to 0.95)	0.002

^a^ SEA-ART scale score: score measured by the Scale of Self-efficacy for Adherence to Antiretroviral Treatment

^b^ Odds ratios were adjusted for age in years, sex, formal education in years and time in treatment in months

^c^ p-value calculated by Independent sample *t*-test for differences in means

^d^ p-value calculated by Likelihood test for linear trends

**Fig 1:**
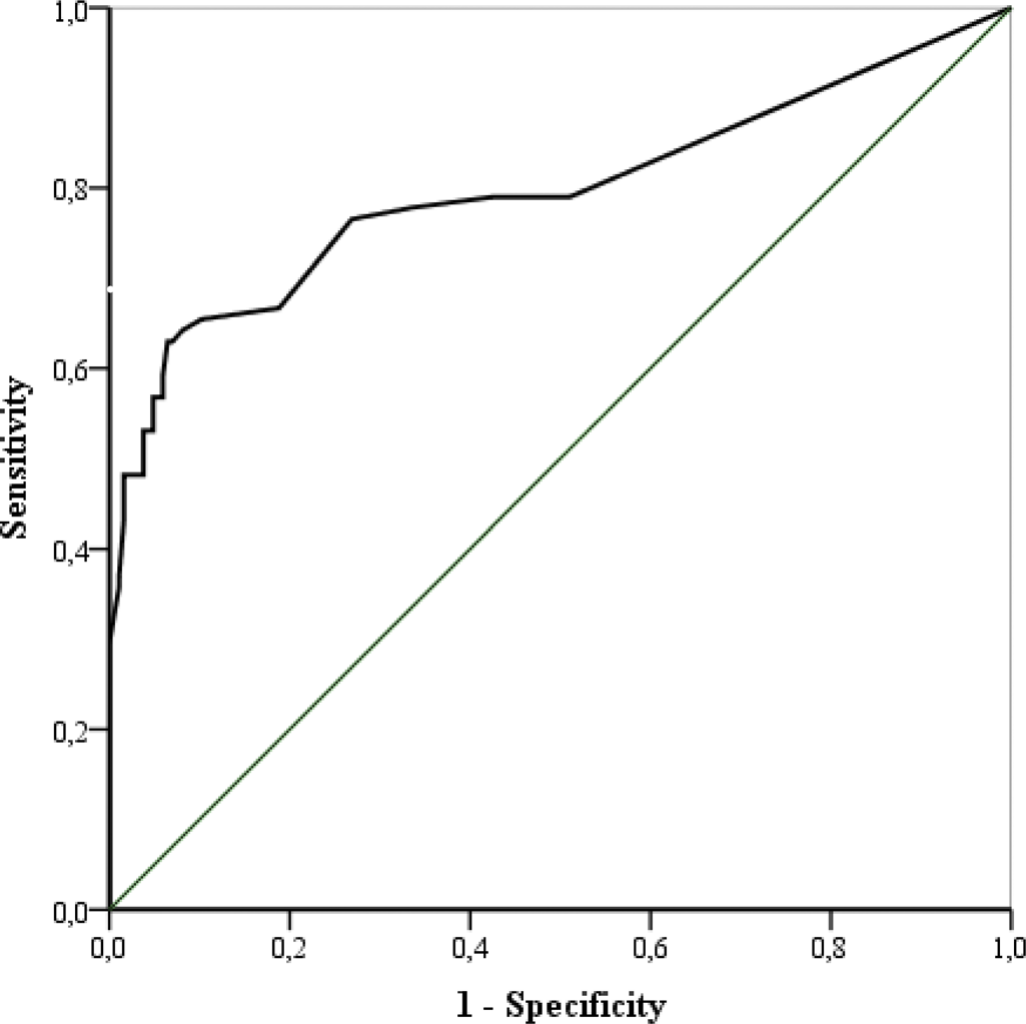
The receiver operating characteristic curve (ROC curve) for non-adherence to ART defined as an intake of less than 95% of the prescribed medications and the score of self-efficacy expectations of adhesion to ART (SEA-ART total score).

The AUROC for the SEA-ART score was 0.80 (95%CI 0.73–0.87; p<0.001) (Fig 1). The cut-off point of 101 units of the SEA-ART score was associated with the highest specificity and sensitivity in predicting non-adherence.

The prevalence of non-adherence and the predictive validity of the SEA-ART cut-off point of 101 units, relative to non-adherence to HIV treatment at one-month follow-up is presented in Table 4 for the overall sample and for groups organized by age, gender, formal education and time in treatment. The cut-off value of 101 provided a sensitivity of 76.5%, a specificity of 73.1%, a positive predictive value of 55.4% and a negative predictive value of 87.7% in detecting non-adherence at one-month follow-up. There was no evidence of difference in the sensitivity, specificity and negative predictive value of the scale among groups of patients organized by age, gender, formal education and time in treatment (all χ^2^, p ≥0.181). The prevalence of non-adherence and the positive predictive value were higher among participants from 18 to 34 years compared to those with 35 and older (χ^2^, p = 0.014 and χ^2^, p = 0.049 respectively). The differences in the prevalence of non-adherence between groups organized by gender, formal education and time in treatment were not statistically significant (χ^2^, p = 0.288, p = 0.065 and p = 0.466 respectively). Similarly, there was no evidence of difference in positive predictive value between groups organized by gender, formal education and time in treatment (all χ^2^, p = 0.503, p = 0.291 and p = 0.674).

**Table 4:** Predictive validity of the SEA-ART score^a^ cut-off point of 101 units relative to non-adherence to HIV treatment at one-month follow-up, by sample characteristics.

Sample variables	Non-adherence %	Sensitivity^b^ %	Specificity^c^ %	PPV^d^	NPV^e^
**Total**	30.3	76.5	73.1	55.4	87.7
	(n = 267)	(n = 81)	(n = 186)	(n = 112)	(n = 155)
**Age (years)**					
18 to 34	37.4	81.6	28.0	63.5	86.8
≥35	23.5	68.8	26.0	44.9	88.5
χ^2^, p-value	0.014	0.181	0.750	0.049	0.743
**Gender**					
Male	32.6	73.2	25.9	57.7	85.1
Female	26.3	84.0	28.6	51.2	92.6
χ^2^, p-value	0.288	0.290	0.686	0.503	0.178
**Education (years)**					
0 to 4	36.9	81.3	69.2	60.8	86.5
≥5	26.2	72.1	75.2	50.8	88.3
χ^2^, p-value	0.065	0.315	0.381	0.291	0.745
**Time in treatment (months)**					
≤ 6	27.0	70.0	75.9	51.9	87.2
>6	31.6	78.7	72.0	56.5	88.0
χ^2^, p-value	0.466	0.426	0.581	0.674	0.899

^a^ SEA-ART score: Score of self-efficacy expectations of adherence to ART.

^b^ Sensitivity is the percentage of SEA-ART score ≤101 among non-adherents to treatment.

^c^ Specificity is the percentage of SEA-ART score >101 among adherents to treatment.

^d^ PPV (Positive predictive value) is the percentage of non-adherence among individuals with self-efficacy score ≤ 101.

^e^ NPV (Negative predictive value) is the percentage of adherence among individuals with SEA-ART score >101.

## Discussion

This study examined the validity of the 21-item SEA-ART scale in predicting non-adherence to ART in a specialised outpatient public service with a catchment area of about 300,000 inhabitants in a metropolitan region in Southern Brazil. The selected sample included all adults who started ART in the three years preceding the study, including service attenders and dropouts. Because outpatient treatment for HIV in Brazil is provided only by specialised public services, the selected sample is likely to reflect short and long term experience of ART in adults in this region.

The participation rate of 86% of the selected sample was relatively high. The sociodemografic characteristics (predominance of men, age group 25 to 44 years and low levels of schooling) and the prevalence of ART adhesion of 30.3% in the effective sample are similar to other studies in the Brazilian population in ART in the last 20 years [19], which suggests that the study results may be applied with caution to the Brazilian population of adults on ART.

Results show patients’ good acceptability of the SEA-ART items and reasonable distribution of item responses. The corrected-item total correlations suggest that all items contribute to the scale. The high internal consistency reliability of the scale (Crombach-α = 0.95) indicates that the SEA-ART items can be combined to produce a scale likely to measure a single construct. Face and content validity analysed by five experts and seven patients in a previous study [11] suggests that the scale assesses patients’ expectations of their own ability to overcome major high-risk situations for non-adherence to ART. The items of the SEA-ART scale were phrased as to assess self-efficacy expectations by examining how confident the individual is in performing the actions to overcome high-risk situations for non-compliance. The items scale cover three areas of personal experience likely to be associated with non-compliance: emotional and physiological states related to negative experiences with treatment and low concern with the illness, unsupportive social relationships, and environmental circumstances and treatment schemes that require attention and organization. Such contents have been considered of paramount importance in HIV/AIDS treatment effectiveness [13,20,21]. The strength of the self-efficacy expectations for adherence to ART varied greatly among the risk situations anticipated by the patients. Low efficacy expectations were more commonly reported for the experience of unwanted effects of the treatment (25.8% of the patients), (b) worries about the disclosure of their HIV condition (12.4%) and (c) when going from one place to another at the time they should take the medication (8.0%). Such findings are in line with previous studies showing that strategies for privacy when taking the HIV medications, self-management and organization of daily life activities are crucial for adherence to HIV treatment [2,22]. Therefore, these results on internal validity suggest that the SEA-ART scale is likely to be a reliable measure of self-efficacy for adherence to antiretroviral treatment for HIV in adults and that the scale has good acceptability among the patients.

The SEA-ART score was shown to predict treatment status at one-month follow-up in different ways. The score on the SEA-ART scale varied from 21 to 105 within the sample. On average, the SEA-ART score was higher for patients who were adherent to treatment compared to the non-adherents (means 101.7 and 76.0; difference in means p<0.001), suggesting that high self-efficacy is needed for ART adherence. The odds of non-adherence was negatively associated with the SEA-ART score, after adjustment for age, sex, education and time in treatment (p = 0.002). On average, the estimated odds of non-adherence was 8% lower for each unit of increase in the SEA-ART score (OR = 0.92). Therefore, if the self-efficacy score is higher by “n” units, the odds of non-adherence is expected to decrease by a factor of 0.92^“n”^. For example, a difference of 4 points in SEA-ART score between two groups of patients imply a 28% difference in the odds of non-adherence (100–0.92^4^ = 0.28). These results suggest that even small increases in self-efficacy expectations in the population may have a positive impact on the prevalence of adherence to HIV treatment.

Results on the ROC curve analysis indicated that the cut-off point of 101 units on the SEA-ART score provides the highest sensitivity and specificity in predicting non-adherence to ART. Sensitivity and specificity are independent of prevalence and reflect the discriminating capability of the SEA-ART scale. Results on sensitivity and specificity show that 76.5% of the non-adherents and 73.1% of the adherents are likely to be identified when the cut point of 101 units is applied as a tool for identifying risk for non-adherence to treatment for HIV. There were no evidence of differences in sensitivity and specificity between groups defined by age, gender, education and time treatment, suggesting that the discrimination capability of the SEA-ART scale is likely to be similar across socio-demographic circumstances and time in treatment.

The probability of correctly identifying adherence status (predictive value) depends on the prevalence of non-adherence and on the SEA-ART scale capability to identify non-adherence (sensitivity), or adherence to ART (specificity). The positive predictive value (probability of correctly identifying non-adherence) increases with the prevalence. The average positive predictive value of 55.4% corresponds to the application of the SEA-ART scale in populations with a prevalence of non-adherence of 30%. The positive predictive value was higher for patients younger than 35 years (63.5%) compared to the others (44.9%), reflecting the greater prevalence of non-adherence in the younger patients. The average positive predictive value of 55.4% implies that about 44.6% of the patients are likely to be misclassified as non-adherent. To avoid that these patients be unnecessarily exposed to treatment adherence interventions, their adherence status should be confirmed by an in-depth interview, similar to a test in series. The negative predictive value of 87.7% is relatively high, but approximately 12% of the patients with high self-efficacy score are likely to report non-adherence to ART at one-month follow-up. This indicates that other methods, such us monitoring antiretroviral medication withdrawal from the pharmacy should be applied to support the follow up of all patients. The negative predictive value appear similar across socio-demographic circumstances and time in treatment, but this should be confirmed in further studies.

The present study has some limitations. *First*, the major objective of the study was to assess the validity of the SEA-ART in predicting non-adherence to ART at one-month follow-up. Adherence in the short term was chosen to support early identification of patients in greater need of psychosocial support. Further studies should examine the predictive validity of the scale in longer follow-up periods. *Second*, the measurement of adherence to ART is another relevant issue in this study. ART adherence (defined by use of ≥ 95% of the ART prescription) was assessed by a detailed 24-hour inventory applied by trained fieldworkers who were unaware of the patient medical prescription. The self-reported adherence was associated with treatment effectiveness measured by undetected viral load (p<0.001). Although self-reported adherence should be treated with caution, these results support the validity of the adherence measurement. Studies applying serum dosages of the antiretroviral drugs must confirm the predictive validity of the SEA-ART scale on ART adherence. *Third*, because the sample size is relatively small and the study was conducted in one clinical population only, further studies with greater study power should examine group differences in sensitivity and specificity of the SEA-ART scale in other clinical populations.

In conclusion, the SEA-ART scale is rapid and simple assessment of patients’ confidence in their ability to cope with ART. The scale appears to be a reliable and valid tool to help predicting adherence status in the short term (within one month) in adults. The cut-of point of 101 units on the SEA-ART scale is associated with reasonable sensitivity and specificity in discriminating adherence status, being potentially useful as a pre-screening of risk for non-adherence to ART in clinical practice and research studies. The positive and negative predictive values depend on the prevalence of non-adherence in each population. We avoid recommending one particular score level of the scale being applied in all cases, because the scale may be applied for different purposes. Health care providers and researchers may select other cut points that make sense for particular applications. For example, a research study on the efficacy of an intervention for patients who are non-adherent to treatment might opt for a cut point with higher specificity to minimize false-positive cases in the trial. In contrast, a disease management program interested in an initial screening tool might choose a cut point with higher sensitivity to ensure that most patients with poor adherence are included in the program. Further studies should examine the results of the application of the scale in these situations. It is anticipated that this scale can play a role in ongoing and future efforts to evaluate and refine ART adherence guidelines and actions.
